# Self-assembling TLR2 agonists promote mucosal immune responses without pulmonary immunopathologic injuries in mice

**DOI:** 10.1038/s41541-025-01185-y

**Published:** 2025-06-18

**Authors:** Zhangping Huang, Caiguanxi Deng, Lin Peng, Liru Shang, Juan Jiang, Wei Yu, Hao Yang, Jing Liu, Liwei Jiang, Teng Zuo, Ji Wang, Xiafeng Wang

**Affiliations:** 1 Department of Laboratory Medicine, The First Affiliated Hospital, Sun Yat-sen University, Guangzhou, China; 2 Institute of Precision Medicine, The First Affiliated Hospital, Sun Yat-sen University, Guangzhou, China; 3 School of Life Sciences, Liaoning University, Shenyang, China; 4 Laboratory of Immunoengineering, Institute of Health and Medical Technology, Hefei Institutes of Physical Science, Chinese Academy of Sciences, Hefei, China

**Keywords:** Vaccines, Adjuvants, Protein vaccines, Applied immunology, Vaccines

## Abstract

Nasal vaccines offer advantages in eliciting mucosal immunity, particularly through the induction of dimeric IgA. However, the complex mucosal environment poses challenges in achieving optimal immunogenicity and safety. This study introduced Diprovocim, a TLR2 agonist, as an effective and safe adjuvant for mucosal vaccines. Our results demonstrated that Diprovocim self-assembled into particles of suitable size for mucosal delivery, enhancing antigen phagocytosis of immune cells in both lymph nodes and lungs. After effectively activating the TLR2 signaling pathway, Diprovocim led to a reduced release of inflammatory cytokines in vivo without any tissue damage or weight loss, highlighting its safety profile. In mice, both intramuscular and intranasal immunization with Diprovocim-adjuvanted vaccines induced high titers of systemic antibodies. Higher IgG and IgA antibodies were detected in bronchoalveolar lavage fluid (BALF). Moreover, Diprovocim enhanced the immunogenicity of ovalbumin (OVA) and recombinant SARS-CoV-2 protein (RFD-Fc) vaccines, achieving higher CD4^+^ and CD8^+^ T cell immune responses and cross-protection against SARS-CoV-2 variants. These findings highlight the potential of self-assembled Diprovocim as a safe and effective adjuvant for mucosal vaccines, offering a promising strategy for combating respiratory infections.

## Introduction

In recent centuries, humanity has confronted a series of respiratory pandemics, including severe acute respiratory syndrome (SARS), influenza, the Middle East respiratory syndrome (MERS), and the COVID-19 pandemic. Viral diseases such as severe acute respiratory syndrome coronavirus 2 (SARS-CoV-2) pose significant threats to global health, causing severe respiratory illness and mortality^1,2^. Lower respiratory tract infections remain a leading cause of morbidity and mortality worldwide, contributing to approximately 2.4 million deaths annually^3^. The mucosa, as the primary barrier against pathogens, plays a pivotal role in host defense. Strengthening the immune response in the respiratory mucosa emerges as a critical strategy for preventing and treating respiratory pandemics. Nasal vaccination has demonstrated the capacity to induce systemic IgG antibodies^4–6^, and offers a promising approach to elicit long-lasting mucosal immunity, including the development of tissue-resident memory T cells, B cells, and IgA^7,8^. IgA, as a secreted dimeric antibody, can directly traverse the respiratory epithelium to neutralize respiratory pathogens^9^. Notably, research has shown that intranasal vaccination conferred the requisite protection for the entire respiratory system without nebulized inhalation^10^.

The majority of nasal vaccines currently under development or clinical trials are either live-attenuated or virus vector-based vaccines^11–13^. However, these vaccine types have inherent safety concerns and tolerability problems, which pose a risk of mild infection and adverse effects^11,14,15^. Subunit or recombinant protein vaccines offer a safer and more precise approach to vaccination, eliciting an immune response without introducing a living organism or nucleic acid^16^. This feature minimizes adverse reactions. Consequently, mucosal vaccination with subunit vaccines becomes more suitable for individuals across all age groups and immunocompromised patients^17–19^. Despite these advantages, a key limitation of subunit vaccines is their relatively low immunogenicity. Moreover, in the respiratory environment, recombinant protein antigens are prone to clearance by various mechanical, cellular, and chemical barriers. Designing the mucosal recombinant protein vaccines necessitates the incorporation of suitable adjuvants to enhance the antigen uptake and vaccination efficacy^20^. Adjuvants approved for use in human vaccines include aluminum hydroxide, MF59, AS01, Matrix-M, AS03, AS04, and CpG 1018^21^. However, their clinical use in mucosal vaccines is also limited. Conventional adjuvants, such as aluminum hydroxide, have been found inadequate in facilitating immunoglobulin class switching to IgA and IgG2a/c^22,23^, as well as in recruiting T and B cells to mucosal tissues^24^. They are not suitable for all antigens, particularly peptide-based antigens^25,26^. Additionally, the use of adjuvants requires stringent safety considerations. For example, substances administered nasally can be transported to the brain via the olfactory bulb, potentially affecting neurological function^27^. Researchers have extensively studied enterotoxins such as cholera toxin (CT) and heat-labile toxin (LT) as potential adjuvants for mucosal vaccinations. They have been tested in clinical trials for oral mucosal vaccinations, like ACE527 and ETVAX, which showed efficacy against Escherichia coli-induced traveler’s diarrhea^28,29^. However, serious safety concerns remain regarding enterotoxin adjuvants. Even small doses of purified CT (as low as 5 μg) can induce severe diarrhea^30^, and LT can induce fluid secretion at doses as low as 2.5 μg^31^. Moreover, evidence suggests that enterotoxins could traverse the central nervous system via the olfactory nerve, potentially leading to severe adverse effects such as Bell’s palsy or facial paralysis in intranasal vaccine recipients^32^. Therefore, new adjuvant strategies are needed to mitigate side effects and ensure the safety of mucosal vaccination.

Protein antigens generally elicit regulatory or inhibitory immune responses that result in tolerance. Microbial components, such as toll-like receptor (TLR) ligands, can trigger robust cellular and humoral immune responses capable of overcoming mucosal tolerance. TLR agonists function similarly to pathogen-associated molecular patterns (PAMPs). These agonists bind to pathogen recognition receptors (PRRs) expressed on antigen-presenting cells (APCs)^33^. Therefore, TLR agonists represent an innovative and promising class of vaccine adjuvants. Unlike other TLRs (TLR3/7/8/9) that recognize intracellular pathogen-derived nucleic acids^34^, TLR2 is expressed on the cell surface, making it a suitable target for recognizing extracellular pathogens^35^. Various cell types within the respiratory system express TLR2, including dendritic cells, macrophages, and respiratory epithelial cells^36,37^. Importantly, some TLR2 agonists exhibit self-adjuvant capacities, meaning they can covalently conjugate with antigens for simultaneous uptake by immune cells^38^, such as Pam2CSK4 and MALP-2^39^. Innate immune receptor ligands enhance the host immune response to antigens by activating innate immunity, effectively triggering acquired immunity. This property enables them to activate T cells, mature dendritic cells, and facilitate the passage of vaccines through physiological barriers in mucosal tissues. The efficacy of TLR2 agonists has been demonstrated in mucosal vaccine studies^40,41^.

**Fig. 1:**
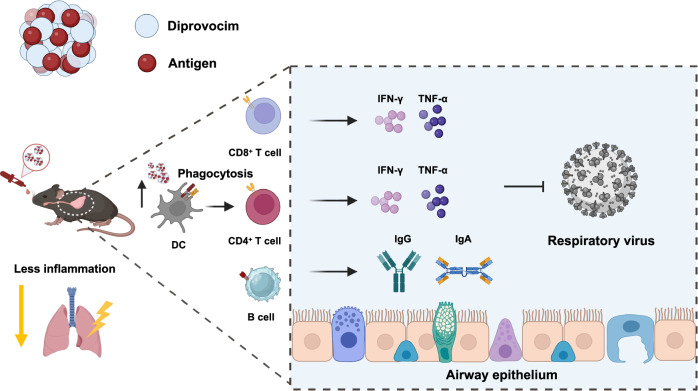
Schematic illustration of self-assembling Diprovocim vaccines promotes mucosal immune responses without pulmonary immunopathologic injuries in mice. Diprovocim self-assembling nasal vaccines enhanced dendritic cell phagocytosis, and induced potent humoral and cellular immune responses while minimizing lung inflammation (Created with BioRender.com).

Diprovocim is a TLR2 agonist that stimulates the NF-κB and MAPK pathways upon activation^39^. Its unique structural composition allows for simple synthesis and modification^42^. Diprovocim, with a low activation concentration (EC_50_ = 110 pM) has shown efficacy as a vaccine adjuvant in preventing viral infections and melanoma via intramuscular injection^43,44^. Importantly, Diprovocim does not induce IFN-β in macrophages, suggesting a favorable safety profile^43^. In addition, previous studies have demonstrated Diprovocim’s self-assembling properties with antigen, which enhance the delivery efficiency of subunit vaccines^44^. In order to explore the feasibility of TLR2 agonists in mucosal vaccination, this study comprehensively evaluated the immunogenicity and safety of Diprovocim for subunit vaccines in vitro and in vivo. Our findings indicated that Diprovocim significantly enhanced the phagocytosis of antigen-presenting cells, thereby facilitating nasal immunization. In murine experiments, Diprovocim induced only mild systemic inflammatory responses while simultaneously eliciting strong systemic and mucosal immune protection. These results suggested that Diprovocim offered a favorable balance of improved immunogenicity and a decent safety profile. Our study highlighted the significant potential of Diprovocim as a nasal adjuvant for the development of vaccines against respiratory infections (Fig. 1).

## Results

### Diprovocim self-assembled with OVA to enhance uptake and activation in vitro

**Fig. 2:**
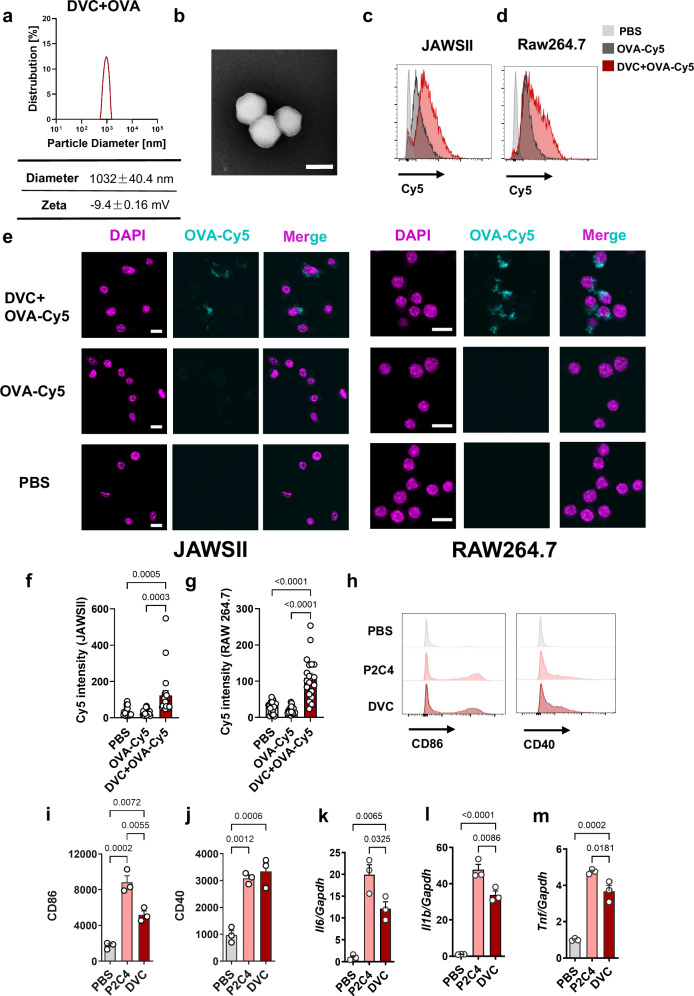
Self-assembling particles enhanced uptake and maturation in vitro. **a** Particle diameters and zeta-potential of 1:1 mixture of Diprovocim and OVA (DVC + OVA) particles. **b** DVC + OVA image examined by SEM. Scale bar, 1 μm. **c**, **d** Confocal microscopy images of intracellular uptake. *n* = 3. **e** Intracellular uptake images acquired by confocal microscopy. Scale bar, 10 μm. *n* = 20. **f**, **g** Analysis of relative fluorescence intensity. *n* = 20. **h–j** CD86 (**i**) and CD40 (**j**) were monitored after treating BMDCs with Pam2CSK4 (P2C4) or Diprovocim (DVC). *n* = 3. **k–m** BMDCs were incubated with P2C4 or DVC. *Il6*, *Il1b*, and *Tnf* were measured by RT-qPCR. *n* = 3. Data are shown as mean ± SEM. Statistical significance was determined using one-way ANOVA with Tukey’s multiple comparisons test (**f**, **g**, **i–m**).

The harsh physical and chemical environment of mucosal surfaces presents a significant challenge for protein mucosal vaccines. An intranasal vaccine formulation must navigate the respiratory cavity and penetrate the mucus layer to facilitate uptake. Particulate carriers offer several advantages in vaccine delivery. These include protection of labile antigens and enhanced delivery through targeting antigen-presenting cells (APCs) or draining lymph nodes, thereby minimizing off-target effects^44^. Due to the high hydrophobicity of Diprovocim, it spontaneously formed particles in PBS (Supplementary Fig. 1a). Moreover, we found the particles successfully formed when mixing Diprovocim and Ovalbumin (DVC + OVA). We investigated the particle diameters resulting from the mixing of Diprovocim and OVA at various ratios. Our findings indicated a correlation between particle diameter and mixing ratio, with higher proportions of Diprovocim resulting in larger particles (Supplementary Fig. 1b). Previous studies have shown that increasing particle size beyond 1 μm leads to greater inertia. This results in particle deposition in the nasal cavity due to Brownian diffusion, which can impact vaccine efficacy^45,46^. Additionally, intranasal administration of particles smaller than 900 nm may be transported to the brain via nerves, raising safety concerns^47,48^. When mixing equal masses of Diprovocim and Ovalbumin (DVC + OVA), particle analysis revealed a diameter of 1032 nm for the resulting Diprovocim+OVA particles (Fig. 2a). Therefore, we chose this ratio to perform the next experiments. Other TLR2 agonists, such as Pam2CSK4 and Pam3CSK4, did not form particles under these conditions. The zeta potential of the particles was measured to be about −9.4 mV (Fig. 2a). Meanwhile, scanning electron microscope (SEM) showed that Diprovocim+OVA was self-assembled into particles (Fig. 2b). Given Diprovocim’s ability to form particles with antigens, we investigated the cellular uptake of these composites in JAWSII (dendritic cells) and RAW264.7 (macrophages) cell lines. We synthesized a fluorescently labeled antigen, OVA-Cy5, mixed it with Diprovocim, and incubated the particles with cells. Flow cytometry analysis revealed stronger Cy5 signals in the Diprovocim group compared to OVA-Cy5 alone in both cell lines (Fig. 2c, d). To further investigate the uptake of particulate antigens by APCs, we performed immunofluorescence staining on both cell lines and quantified Cy5 uptake. Immunofluorescence staining revealed significantly higher mean fluorescent intensity in the Diprovocim group compared to OVA-Cy5 alone in both JAWSII and RAW264.7 cells (Fig. 2e). These results were confirmed by statistical analysis (Fig. 2f, g). We compared the effects of Diprovocim with Pam2CSK4, a well-established TLR2 agonist, which is known for its potent adjuvant properties in nasal vaccination^49,50^. We measured the expression of co-stimulatory factors CD40 and CD86 on bone marrow-derived dendritic cells (BMDCs) after 24-h incubation with Diprovocim or Pam2CSK4 by flow cytometry. Both Diprovocim and Pam2CSK4 significantly upregulated CD86 and CD40 expression compared to PBS. Diprovocim induced a 3.0-fold increase in CD86 expression and a 3.2-fold increase in CD40 expression (Fig. 2h–j). To further investigate the activation potential and inflammatory profile of Diprovocim, we performed quantitative real-time PCR (RT-qPCR) to assess the downstream signaling pathways following TLR2 activation. TLR2 activation triggers the activation of the transcription factor NF-κB, which plays a crucial role in the transcriptional regulation of pro-inflammatory cytokines such as TNF-α, IL-1β, and IL-6. Diprovocim induced the expression of these cytokines but to a lesser extent than Pam2CSK4 (Fig. 2k–m). These results suggested that Diprovocim effectively activated APCs.

### Diprovocim enhanced phagocytosis in MLNs and lungs

**Fig. 3:**
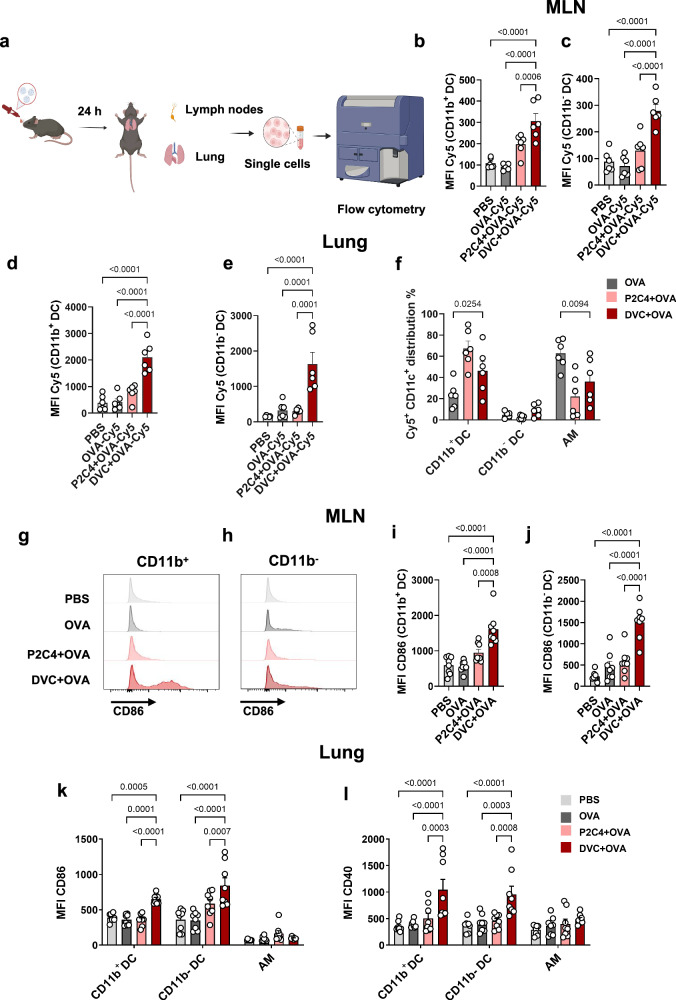
Diprovocim enhanced uptake and maturation in vivo. **a** Schematic illustration of Diprovocim as a nasal adjuvant enhancing antigen uptake and immune cell activation 24 h post nasal immunization (Created with BioRender.com). **b**, **c** Cellular uptake of OVA-Cy5 in different cell types of Mediastinal lymph nodes (MLNs). *n* = 6. **d**, **e** Cellular uptake of OVA-Cy5 in different cell types of lungs. *n* = 6. **f** Cell distribution of Cy5^+^ CD11c^+^ cells within the lung tissue. *n* = 6. **g–j** CD86 in different cell types of MLNs was monitored after nasal injection with Pam2CSK4 or Diprovocim 24 h post-nasal immunization. *n* = 8. CD86 (**k**) and CD40 (**l**) on different cell populations in the lungs were monitored after 24 h by nasal immunization. *n* = 8. Data are shown as mean ± SEM. Statistical significance was determined using one-way ANOVA with Tukey’s multiple comparisons test (**b–e**, **i**, **j**) or two-way ANOVA with Tukey’s multiple comparisons test (**f**, **k**, **l**).

To further investigate Diprovocim’s impact on antigen delivery, we examined the uptake of intranasally administered OVA-Cy5 mediastinal lymph nodes (MLNs) and lung cells in mice (Fig. 3a). Uptake was assessed both with and without Diprovocim or Pam2CSK4. We analyzed the mean fluorescence intensity (MFI) and percentage of Cy5^+^ cells in different cell populations, with the gating strategy outlined in Supplementary Fig. 2. DCs are the primary APCs in lungs that cross-present antigens to CD8^+^ T cells during viral infections. They migrate to lymph nodes for antigen presentation and T cell activation^48^. Our results revealed a significant increase in Cy5 signal intensity in all CD11c^+^ DC subsets in the MLNs, including CD11b^-^ and CD11b^+^ DCs, when co-administered with Diprovocim. CD11b^+^ DCs are known to induce robust antiviral CD8^+^ T cell and humoral immune responses^48^. CD11b^-^ DCs can cross-present antigens from virus-infected cells due to their resistance to viral infection through a type I interferon-mediated antiviral state^51^. Both DC subsets play crucial roles in combating infections. In the MLNs, Diprovocim significantly increased the uptake of OVA-Cy5, particularly in CD11b^-^ DCs. In CD11b^+^ DC, Diprovocim increased uptake by 1.6-fold compared to the Pam2CSK4 group (Fig. 3b). The MFI of Cy5 in CD11b^-^ DCs was ~2.2- and 3.9-fold higher in the Diprovocim group compared to the Pam2CSK4 and OVA-Cy5 groups, respectively (Fig. 3c). A similar trend was observed for the percentage of Cy5^+^ DCs (Supplementary Fig. 3a–d). There was no significant difference in the percentage of CD11c^+^ DCs among all groups in the MLNs (Supplementary Fig. 4a). Following intranasal administration of OVA, few CD11b^+^ or CD11b^-^ DCs in the lung exhibited OVA uptake. However, in the presence of Diprovocim, the MFI of Cy5 in CD11b^+^ and CD11b^-^ DCs both obviously increased compared to the OVA group (Fig. 3d, e). The percentage also showed the same trend (Supplementary Fig. 5a–d). Diprovocim demonstrated a strong enhancement of phagocytosis in both lymph nodes and lungs, whereas Pam2CSK4 showed no significant increase in fluorescence. After analysis of the distribution of Cy5^+^ CD11c^+^ cells in the lung, we found that in the two adjuvant groups, OVA-Cy5 was primarily engulfed by CD11b^+^ DC. Diprovocim enhanced the MFI of Cy5, targeting CD11b^+^ DCs, which constituted 46.5% of the Cy5^+^ cell population (Fig. 3f). These results indicated that Diprovocim enhanced antigen uptake in MLNs and lungs.

### Diprovocim self-assembling particles activated immune cells via the nasal route

Next, we evaluated whether antigen-Diprovocim self-assembly particles could enhance immune cell activation and maturation in vivo. After 24 h of nasal injection of OVA, Diprovocim significantly enhanced CD86 expression in MLNs (Fig. 3g–j). Specifically, CD86 expression was enhanced 2.9-fold in CD11b^+^ DCs and 3.2-fold in CD11b^-^ DCs compared to the OVA-only group. Moreover, Diprovocim-induced CD86 expression was 1.7- and 2.6-fold higher than that observed with Pam2CSK4. A similar upregulation was observed in the percentage of CD86^+^ cells (Supplementary Fig. 3e–h). Total CD11c^+^ cells in MLN exhibited the highest CD86 MFI and percentage (Supplementary Fig. 4b–e). In the lung, flow cytometry analysis revealed that both resident CD11b^+^ and CD11b^-^ DCs exhibited increased CD86 and CD40 expression in the Diprovocim group (Fig. 3k, l). The percentage demonstrated the same results (Supplementary Fig. 7a, b). Alveolar macrophages (AMs), however, showed no significant change. In contrast, Pam2CSK4 failed to induce a significant increase in DC activation markers. These findings highlighted the unique and potent immunostimulatory properties of Diprovocim compared to Pam2CSK4, particularly its ability to induce immune cell maturation and activation in the lung microenvironment. This underscores the potential of Diprovocim as a promising adjuvant for enhancing immune responses.

### Diprovocim showed less inflammation and tissue damage

**Fig. 4:**
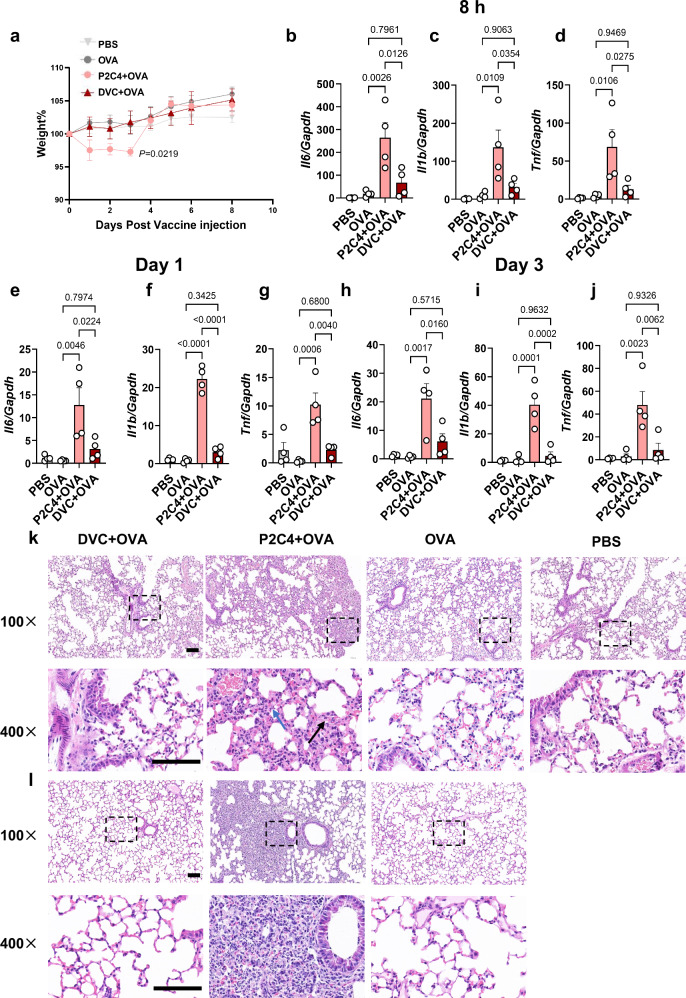
Diprovocim elicited a reduced inflammatory profile without pulmonary immunopathologic injuries. **a** Body weight changes were monitored for 8 days post-immunization. *n* = 4. **b–j** Relative mRNA expression levels of *Il6*, *Il1b*, and *Tnf* in lung tissue were assessed at various time points post-nasal vaccination by RT-qPCR. *n* = 4. **k**, **l** Hematoxylin and eosin (H&E) stained histological images of lung tissue. Mice were sacrificed 1 day (**k**) or 3 days (**l**) post-nasal immunization with PBS, OVA, P2C4 + OVA, and DVC + OVA. Lymphocyte infiltration (blue arrow), fibroplasia (black arrow). Scale bar, 100 μm. Data are shown as mean ± SEM. Statistical significance was determined using two-way ANOVA with Dunnett’s multiple comparisons test (**a**) or one-way ANOVA with Tukey’s multiple comparisons test (**b–j**).

Given the heightened safety concerns associated with adjuvants in mucosal vaccines, we evaluated the effect of Diprovocim to induce systemic adverse effects or inflammatory responses following mucosal administration. Mice immunized with Diprovocim+OVA showed no change in body weight, whereas Pam2CSK4+OVA experienced a significant 2.8% weight loss by 3 days post-immunization (Fig. 4a). To assess inflammatory responses, we measured cytokine levels in the lung at different time points following nasal immunization. TNF-α, IL-1β, and IL-6 are major pro-inflammatory cytokines involved in the acute inflammatory response. Diprovocim induced only slight increases in cytokine levels, with no significant differences compared to the OVA-only group at 8 h, day 1, or day 3 post-immunization (Fig. 4b–j). In contrast, Pam2CSK4 elicited a more pronounced and rapid inflammatory response at 8 h after immunization, characterized by significantly elevated levels of pro-inflammatory cytokines. Even on the third-day post-immunization, Pam2CSK4-treated mice exhibited a 4-fold, 7.4-fold, and 4.4-fold increase in *Il6*, *Il1b*, and *Tnf*, respectively, compared to the Diprovocim group (Fig. 4h, i). Consistent with the cytokine data, histological analysis of lung tissue sections revealed inflammatory changes (Fig. 4k), with more severe pulmonary inflammation observed in the Pam2CSK4 group on day 3 (Fig. 4l). We replicated the experiments regarding weight loss and cytokine level detection, and the results exhibited the same trend as well (Supplementary Fig. 8). In the lungs, multiple inflammatory infiltrates were observed. These included fibroplasia in subserosal and peribronchial areas, blurred alveolar structures, merging alveolar cavities, and infiltration by inflammatory cells. In contrast, no significant lung damage or inflammation was observed in the Diprovocim or control groups. We also performed a histological analysis of all groups. The results showed a greater degree of eosinophil infiltration, epithelium damage, and necrosis in the Pam2CSK4 group on both day 1 or day 3 (Supplementary Table 1). Overall, Diprovocim offered a safer alternative to Pam2CSK4, avoiding the severe inflammatory reactions and tissue damage observed with the latter.

### Diprovocim was a potent adjuvant for adaptive immune response

**Fig. 5:**
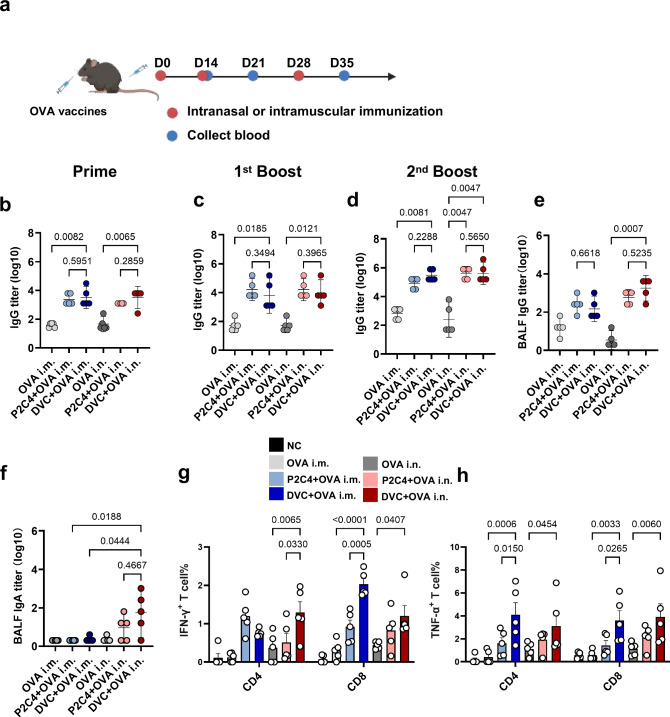
Self-assembling particles vaccination enhanced systemic and mucosal immune responses. **a** Schematic illustration of the nasal and intramuscular immunization and sample collection strategies (Created with BioRender.com). C57BL/6 mice were vaccinated with different OVA vaccines via intramuscular (i.m.) or intranasal (i.n.) routes. **b–d** OVA-specific IgG antibody titers in serum on day 14, day 21, and day 35 post-immunization. *n* = 5. **e**, **f** OVA-specific IgG and IgA antibody titers in bronchoalveolar lavage fluid (BALF) on 7 days after the final immunization. *n* = 5. **g**, **h** Percentage of IFN-γ^+^ and TNF-α^+^ CD4^+^ and CD8^+^ T cells in PBMCs on day 21. *n* = 5. Data are shown as geometric mean ± 95% CI or mean ± SEM. Statistical significance was determined using Kruskal-Wallis test (**b–f**) or two-way ANOVA with Tukey’s multiple comparisons test (**g**, **h**).

The above results demonstrated that Diprovocim was effective in activating APCs in the lung, while inducing fewer inflammatory cytokines and a less severe inflammatory response. Next, we examined its potential as a vaccine adjuvant. In this study, C57BL/6 mice received three homologous immunizations at two-week intervals, administered either intramuscularly (i.m.) or intranasally (i.n.) with OVA protein in the presence or absence of adjuvant (Fig. 5a). Humoral immune responses were assessed after each vaccination. We compared unvaccinated mice to those receiving OVA alone or with Pam2CSK4 or Diprovocim. After three doses, vaccination with Diprovocim, by either route, induced potent anti-OVA IgG titers in serum and bronchoalveolar lavage fluid (BALF). Notably, OVA alone was insufficient to elicit significant humoral immune responses in most animals, regardless of the route of administration or the frequency of immunizations (Fig. 5b–d). Diprovocim had a significant impact, particularly after the primary immunization. Intranasal administration of Diprovocim raised OVA-specific total IgG titers in serum 72.1-fold over OVA alone and 2.6-fold over Pam2CSK4. Intramuscular injection of Diprovocim resulted in a 1.4-fold higher geometric mean titer than Pam2CSK4 (Fig. 5b). After three doses, intranasal administration of Diprovocim induced a 1641.6-fold increase in total IgG titers compared to intranasal OVA alone and comparable levels with Pam2CSK4 (Fig. 5d). Intramuscularly, Diprovocim elicited 3.6-fold higher IgG titers than Pam2CSK4. Notably, intranasal immunization with Diprovocim resulted in a 12.1-fold increase in total IgG titers in BALF compared to intramuscular immunization (Fig. 5e). Additionally, anti-OVA IgG levels were also higher in nasally immunized mice compared to those receiving OVA alone or Pam2CSK4. Surprisingly, anti-OVA IgA was detected only in the serum and BALF of intranasally immunized mice. In these mice, IgA levels were increased 32-fold in serum and 9-fold in BALF compared to mice receiving OVA alone or Pam2CSK4 (Fig. 5f). The generation of systemic T-cell response was assessed by intracellular cytokine staining. One week after the second vaccination with Diprovocim, immunized mice had an of IFN-γ^+^ and TNF-α^+^ T cells, including both CD4^+^ and CD8^+^ T cells (Fig. 5g, h). The percentage of these cells was slightly higher than in the Pam2CSK4 group. Notably, nasal immunization with Diprovocim significantly increased the population of OVA-specific CD4^+^ T cells producing IFN-γ compared to Pam2CSK4. Additionally, Diprovocim significantly enhanced the percentage of TNF-α^+^ OVA-specific CD4^+^ and CD8^+^ T cells with both nasal and intramuscular immunization compared to OVA alone. These findings demonstrated that Diprovocim effectively enhanced both humoral and cellular immune responses, suggesting that robust adaptive immunity can be achieved via the airway.

### Diprovocim induced potent immune response adjuvanted with RBD-Fc

**Fig. 6:**
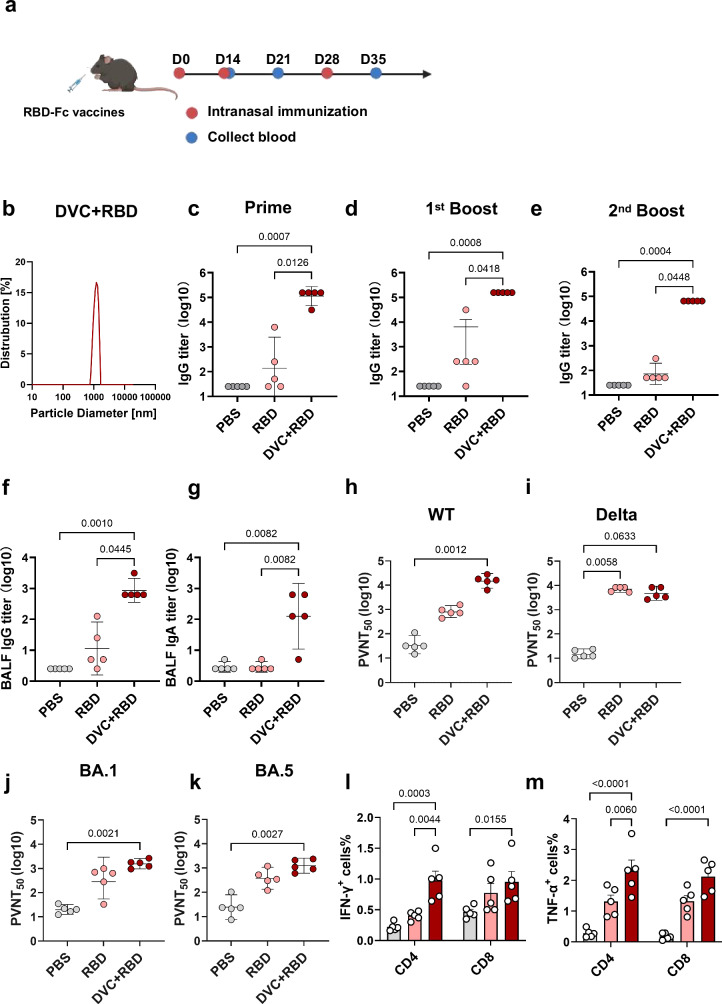
Self-assembling particles nasal vaccination induced potent immune responses and pseudovirus neutralization. **a** Schematic illustration of nasal immunization and sample collection strategies (Created with BioRender.com). C57BL/6 mice were i.n. vaccinated with RBD-fc with or without Diprovocim. **b** Particle diameter and distribution of 1:1 mixture of Diprovocim and RBD-Fc (DVC + RBD). **c–e** RBD-specific IgG antibody titers in serum on day 14, day 21, and day 35 post-immunization. *n* = 5. **f**, **g** RBD-specific IgG and IgA antibody titers in BALF 7 days after the final immunization. *n* = 5. **h**, **i** Pseudovirus neutralization titers (PVNT_50_) in serum, measured by pseudovirus neutralization assay. *n* = 5. **l**, **m** Percentages of the proportion of IFN-γ^+^ and TNF-α^+^ CD4^+^ T cells and CD8^+^ T cells of PBMCs on day 21 post-immunization. *n* = 5. Data are shown as geometric mean ± 95% CI or mean ± SEM. Statistical significance was determined using Kruskal-Wallis test (**c–k**) or two-way ANOVA with Tukey’s multiple comparisons test (**l**, **m**).

Adjuvants play a critical role in inducing sufficient immune responses when recombinant protein vaccines are used to combat respiratory infectious diseases such as SARS-CoV-2. The receptor-binding domain (RBD) is a primary target for neutralizing antibodies (NAbs) and offers significant advantages for vaccine development due to its immunogenic properties^52^. To simulate potential clinical applications of Diprovocim, we used RBD-Fc, a subunit vaccine derived from the Delta variant. RBD-Fc fuses the RBD of SARS-CoV-2 to the Fc region of the human IgG1 antibody. RBD-Fc was administered intranasally three times, both in the presence and absence of Diprovocim (Fig. 6a). After optimizing the ratio of Diprovocim and RBD proteins, mixing Diprovocim with RBD-Fc with 1:1 ratio (referred as DVC + RBD) resulted in particles of 1 μm diameter (Fig. 6b). Notably, intranasal administration of Diprovocim+RBD-Fc induced a robust humoral immune response after the primary immunization, with anti-RBD IgG titers reaching 10^5^ (Fig. 6c). While RBD-Fc alone could induce RBD-specific IgG, the titers were 824.8-fold lower than those achieved with Diprovocim+RBD-Fc. Following the second immunization, Diprovocim+RBD-Fc elicited a significantly more robust response, boosting titers 377.1-fold compared to the response to RBD-Fc alone (Fig. 6d). After two immunizations, Diprovocim continued to enhance anti-RBD IgG titers. Unexpectedly, anti-RBD IgG titers did not increase significantly after the third dose of RBD-Fc, either alone or with Diprovocim (Fig. 6e). We further evaluated antibody responses in BALF one week after the final vaccination (Fig. 6f, g). Anti-RBD IgG titers in BALF were 75.4-fold higher in the Diprovocim+RBD-Fc group compared to RBD-Fc alone. Additionally, anti-RBD IgA levels were significantly increased by 43.5 times compared to RBD-Fc. These results demonstrated Diprovocim’s significant potential as a vaccine adjuvant for respiratory virus infections. It induced high serum IgG titers after the first immunization and enhanced respiratory protection, as evidenced by elevated BALF antibody titers. To assess the neutralizing potential of these antibodies, we performed the pseudovirus neutralization assay 7 days after the last immunization. While RBD-Fc alone generated substantial titers of neutralizing antibodies (NAbs) against the Delta variant, Diprovocim did not significantly increase titers over RBD-Fc (Fig. 6i). However, the cross-neutralizing antibody response induced by RBD-Fc alone against the WT strain was weaker compared to the response induced by Diprovocim+RBD-Fc (Fig. 6h). Remarkably, Diprovocim exhibited a notable adjuvant effect, boosting cross-neutralizing antibody titers by 18.5-fold. Against the more mutated Omicron BA.1 and BA.5 variants, which are known to be more immune-invasive, cross-neutralizing antibody titers induced by RBD-Fc alone were even lower. In contrast, intranasal administration of RBD-Fc with Diprovocim still induced significant cross-neutralizing antibodies against both BA.1 and BA.5 variants compared to PBS (Fig. 6j, k). Furthermore, to determine whether the self-assembly of Diprovocim with proteins affected the epitopes of the proteins, we evaluated the binding affinity of monoclonal antibodies (mAbs) to the RBD-Fc using biolayer interferometry (BLI). We chose two antibodies, including P2C-1F11 and COV2-2196, which both neutralize the virus by blocking RBD access to the human receptor ACE2^53^^,54^. We then investigated their binding to RBD-Fc alone or in a mixture with Diprovocim. The results indicated that RBD-Fc, when assembled into particles with Diprovocim, retained the ability to bind mAbs. P2C-1F11 bound to RBD with a KD of 0.27 nM, whereas its affinity for DVC + RBD was slightly lower (KD = 0.82 nM). Likewise, COV2-2196 demonstrated strong binding to both RBD (KD = 0.17 nM) and DVC + RBD (KD = 0.59 nM) (Supplementary Fig. 10a–c), suggesting that the self-assembly process did not change or mask the epitopes of the RBD. To further investigate the cellular responses, we assessed the cellular immune response in the PBMCs 7 days post-secondary immunization using intracellular cytokine staining. The results further confirmed that Diprovocim+RBD-Fc significantly enhanced the CD4^+^ T cell response more than RBD-Fc priming against SARS-CoV-2. Cytokine productions in the Diprovocim group were higher than the responses elicited by RBD-Fc (Fig. 6l, m). The Diprovocim booster elevated IFN-γ and TNF-α secretion, indicating enhanced cellular immunity. This outcome indicated superior cellular immune responses following nasal vaccination with Diprovocim as an adjuvant. The percentage of IFN-γ^+^ and TNF-α^+^ CD4^+^ T cells was significantly higher in the Diprovocim-adjuvanted group. Similarly, IFN-γ^+^ and TNF-α^+^ CD8^+^ T cells were increased 1.2-fold and 1.6-fold compared to the RBD-Fc group, respectively. Overall, these findings suggested that intranasal immunization with the Diprovocim and RBD-Fc combination enhanced the induction of potent cross-protective effects and cellular responses against SARS-CoV-2, highlighting its potential as a versatile vaccine strategy.

## Discussion

Respiratory viruses primarily transmit through the respiratory tract and replicate efficiently in the trachea and lungs^55^. Controlling infections and pathogen transmission in the respiratory tract is crucial, requiring a strong antiviral immune response in the airways. Parenteral administration methods often fall short of providing comprehensive protection^56^. Significant amounts of viral RNA can persist in the lungs despite traditional vaccinations, whereas a single intranasal immunization has been shown to provide complete protection in both the upper and lower respiratory tracts^10^. Intranasal vaccination can reduce viral shedding and transmission^46^. For nasal vaccines, one of the primary challenges is their relatively low immunogenicity, particularly when using protein-based antigens. To enhance the immunogenicity, appropriate adjuvants should be incorporated into vaccines. However, identifying agonists that balance high immunogenicity with acceptable safety profiles has proven challenging. As a result, no adjuvants have yet been approved for use in nasal vaccines by the FDA. TLR2 plays a crucial role in the immune response to infectious diseases like COVID-19. The spike protein of SARS-CoV-2 can stimulate an inflammatory response through the TLR2-dependent pathway^57^. This suggests that activating TLR2 in nasal vaccines may aid in combating SARS-CoV-2 infection. In this study, we found that the TLR2 agonist Diprovocim demonstrated a favorable balance of enhanced immunogenicity and acceptable safety for mucosal vaccines.

Particulate mucosal vaccines can enhance antigen stability by protecting them from proteolytic degradation and improving cellular targeting. Additionally, these formulations may augment antigen uptake by APCs^57^. In this study, we found that Diprovocim self-assembled with antigens through π-π interactions with tyrosine and phenylalanine residues, or through hydrogen bonding with other residues. This self-assembly characteristic effectively enables protein encapsulation. We then investigated how the ratio of Diprovocim and protein influenced particle size (Supplementary Fig. 1b). Our goal was to achieve a balance between safety and practicality in nasal vaccine development. Studies have shown that intranasal delivery of particles with a diameter of 500–1000 nm can reduce systemic absorption^58^. Olfactory sensory neurons are capable of permitting particles with a size of less than 700 nm to penetrate^47^. On the other hand, particles ranging from 1 to 5 μm are considered optimal for lung delivery, as larger particles may deposit in the throat, while smaller particles may be exhaled^59,60^. However, in the nasal passages of rhesus monkeys, the estimated deposition fraction was approximately 1% for 1 µm particles and increased with larger particle sizes^61^. Considering these safety and efficacy factors, we concluded that Diprovocim self-assembling particles with a diameter of approximately 1 μm were suitable for nasal vaccination (Fig. 2a). After self-assembly with protein antigens, Diprovocim significantly enhanced phagocytosis by DCs in the MLNs and lungs, similar to previous findings^44^. Notably, Cy5 fluorescence intensity increased in both CD11b^-^ and CD11b^+^ DCs following Diprovocim treatment, elucidating the targeting effect on these critical immune cells. The enhanced activation and maturation of DC markers such as CD40 and CD86 further illustrated Diprovocim’s superior potency relative to the positive control Pam2CSK4. CD11b^-^ DCs and CD11b^+^ DCs are crucial for pathogen clearance^60,62^. Our results illustrated that CD11b^+^ DC was the primary phagocytic cell population in the lungs, while also noting a slight increase in Cy5^+^ CD11b^-^ DC distribution. The absence of increased CD11c^+^ DC numbers in MLNs (Supplementary Fig. 4a) suggested that the observed enhancement in fluorescence intensity was attributable to improved DC targeting and phagocytosis. Our results indicated that Diprovocim enhanced AM phagocytosis (Supplementary Fig. 6a–c), which is also crucial for lung defense and infection prevention^63,64^. Self-assembling properties can enhance the solubility, bioavailability, and controlled release of insoluble drugs, facilitating targeted drug delivery^65^. Our results aligned with previous findings indicating that antigen delivery via self-assembled particles can enhance immune cell phagocytosis, thereby improving antigen processing efficiency by APCs^65,66^. Some nanoparticles with adjuvant effects can also exert self-adjuvant effects^67^. Although Diprovocim is not covalently conjugated to the protein, the particle formed by their combination allowed it to simultaneously deliver antigen to immune cells and exert self-adjuvant effects.

However, safety problems remain a concern for adjuvants. Concurrently, Diprovocim did not result in weight loss, indicating a minimal impact on the overall health status of the mice. TLR2 activation is implicated in the acute inflammatory response following acute lung injury (ALI)^68,69^. We observed remarkably lower levels of cytokine mRNA in lung tissues at 8 h, 1 day, and 3 days post-immunization with Diprovocim compared to Pam2CSK4 (Fig. 4b–j). While the Pam2CSK4 group exhibited substantial inflammatory cell infiltration and interstitial pneumonia on the third day, the Diprovocim group showed no pathological damage. The excessive, unregulated TLR2 activation may paradoxically lead to more severe acute lung injury and potentially result in diseases such as septic shock^70^. It has been documented that the polylysine sequence in Pam2CSK4 is toxic^71^, despite its effective induction of antibodies in mucosal vaccines^49^. Therefore, the selection of a safe and effective adjuvant for respiratory mucosal vaccines is crucial. In our studies, compared to Pam2CSK4, Diprovocim demonstrated safety as a mucosal vaccine adjuvant.

Next, we established that Diprovocim functioned as a potent mucosal vaccine adjuvant in murine immunization models (Fig. 5). Diprovocim elicited systemic IgG antibody responses in plasma comparable to those achieved via intramuscular administration in OVA immunization experiments. High antibody titers persisted in the adjuvant groups 60 days post-secondary immunization, demonstrating the potent potential of TLR2 agonists as vaccine adjuvants (Supplementary Fig. 9). Additionally, the local IgG and IgA antibody levels in BALF were higher following nasal administration than intramuscular immunization with Diprovocim. Conversely, intramuscular administration failed to induce detectable antibodies in BALF. Nasal administration of OVA or Pam2CSK4 resulted in lower serum antibody titers compared to Diprovocim nasally. Secreted dimeric IgA has been shown to possess the most effective neutralizing activity against viral infections, particularly during the early stages of infection^72,73^, and offers broader cross-protection than IgG. Furthermore, elevated levels of mucosal IgA are associated with a 65% reduction in the risk of breakthrough infections. The increase in antibody levels in BALF suggested enhanced protection of the lower respiratory tract. This is particularly significant as lower respiratory infections remain a major global health concern, ranking as the fifth leading cause of death worldwide^74^. Children under five years of age are disproportionately affected by these infections, experiencing high mortality rates^75^. Similarly, another TLR2 agonist, Pam2Cys, induces stronger antibody titers via mucosal immunization compared to subcutaneous immunization^40^. This suggests that the mucosal route may be preferred for TLR2 agonists to activate immune pathways. In our study, the high-titer antibodies generated in response to the SARS-CoV-2 model exhibited neutralizing activity against variants including WT, Delta, BA.1, and BA.5, underscoring the capacity of Diprovocim to enhance the immunogenicity of RBD-Fc antigens (Fig. 6h–k). In another research, nasal injection of RBD or RBD-Fc induced lower titers reaching up to only 10^2^ against Omicron variants^76^. In BA.1 and BA.5 strains of SAR-CoV-2, Diprovocim elicited 5.5-fold and 3.4-fold over RBD-Fc. Notably, BA.5 exhibits a strong immune evasion potential and is antigenically distant from BA.1 to BA.2 strains^77^. Such cross-protection is vital for safeguarding high-risk populations, particularly during the emergence of highly pathogenic and mutating viruses, as it may effectively curtail viral transmission during pandemics^78^. We also confirmed that the self-assembling processes of Diprovocim and antigen did not affect the neutralization epitopes of RBD. The formation of particles was primarily attributed to Diprovocim’s strong hydrophobic interactions. Additionally, the multiple benzene rings in Diprovocim could form π–π interactions with tyrosine and phenylalanine residues on the protein, thereby facilitating the encapsulation of the protein. Diprovocim promoted cellular immunity by enhancing the secretion of various cytokines and the activation of CD8^+^ and CD4^+^ T cells, including IFN-γ^+^ and TNF-α^+^. Studies have demonstrated that vaccine-induced protection against SARS-CoV-2 relies on an IFN-driven cellular immune response^79^. CD8^+^ T cells function as effector T cells by secreting the cytokines IFN-γ and TNF-α to fight the virus. Even in B-cell-deficient mice, vaccination protects against infection through IFN-γ-secreting CD8^+^ T cell responses^79^. Inducing systemic and tissue-resident CD4^+^ T cells may be crucial for sustained protection, as they exhibit prolonged survival in tissues^80^. The induction of CD4^+^ and CD8^+^ T cells has been shown to be central to effective T cell immunity in infectious diseases. Our research indicated that Diprovocim exerted a potent adjuvant effect on cellular immunity, as evidenced by higher cytokine secretion.

The development of mucosal vaccines for respiratory diseases such as COVID-19 and influenza is ongoing^11,12,81–86^. However, there are currently no protein-based or subunit vaccines with adjuvants approved for use. This emphasizes the need for further research into the optimization of adjuvants for nasal vaccines. In conclusion, our findings provide comprehensive support for Diprovocim as a promising and safe nasal adjuvant, demonstrating less inflammation and effectively inducing both humoral and cellular immunity to prevent respiratory infectious diseases without pulmonary immunopathologic injuries.

## Methods

### Cell lines

JAWSII and RAW264.7 were maintained in our laboratory, and cultured in RPMI-1640 medium (Corning, 10-040-CVRC) supplemented with 100 U/ml penicillin, 100 μg/ml streptomycin (HyClone, SV30010) and 10% heat-inactivated fetal bovine serum (FBS, ExCellBio, FSP500).

### Mice

C57BL/6 mice were obtained from animal facility of the First Affiliated Hospital of Sun Yat-sen University. All the animal care and experimental procedures were performed with ethical compliance and approval by IEC for Clinical Research and Animal Trials of the First Affiliated Hospital of Sun Yat-sen University.

### BMDCs culture

Bone marrow cells were obtained from tibiaes and femurs of 4 to 6-week-old C57BL/6 mice. In brief, bone marrow cells were cultured with 20 ng/ml of murine granulocyte-macrophage colony-stimulating factor (mGM-CSF, BioLegend, 576306) at the concentration of 1 × 10^6^ /ml in complete RPMI-1640 (supplemented with 100 U/ml penicillin, 100 μg/ml streptomycin and 10% FBS) for 7 days at 37 °C, 5% CO2.

### BMDCs activation

To evaluate the activation markers, 5 × 10^5^ BMDCs were pulsed with 1 μM Diprovocim or 100 ng/ml Pam2CSK4 for 24 h. BMDCs were then harvested and stained by FITC-anti-CD11c (1:100, BioLegend, 117305), PB-anti-CD86 (1:100, Biolegend, 105021), and PE-anti-CD40 (1:100, BioLegend, 157505) for 30 min. The stained samples were examined by flow cytometry (BD, FACS LSRFortessa) and analyzed by FlowJo v10.8. A detailed gating strategy is shown in Supplementary Fig. 2.

### RT-qPCR for cytokine detection

2 × 10^5^ BMDCs were pulsed with 1 μM Diprovocim or 100 ng/ml Pam2CSK4 for 4 h. C57BL/6 were immunized nasally with 30 μg OVA protein plus 30 μg Diprovocim or 10 μg Pam2CSK4 for 24 h. Total RNA from BMDCs and lung tissues was extracted with RNA preparation kit (Axygen, AP-MN-MS-RNA-250) at the indicated time. The RNA was reverse transcribed (Thermo Fisher, K1691) and amplified by real-time PCR using a TB Green Premix Ex Taq II (Tli RNaseH Plus) (TaKaRa, RR820Q) by the QuantStudio Real-Time PCR Detection System (Thermo Fisher). Glyceraldehyde 3-phosphate dehydrogenase (*Gapdh*) served as an internal control. The primers for detection in the assay were listed below:

*Gapdh*-F: ATCAAGAAGGTGGTGAAGCA; *Gapdh*-R: AGACAACCTGGTCCTCAGTGT;

*Tnf*-F: CTGAACTTCGGGGTGATCGG; *Tnf*-R: GGCTTGTCACTCGAATTTTGAGA;

*Il-1b*-F: GAAATGCCACCTTTTGACAGTG; *Il-1b*R: TGGATGCTCTCATCAGGACAG；

*Il-6*-F: TACCACTTCACAAGTCGGAGGC; *Il-6*-R: CTGCAAGTGCATCATCGTTGTTC.

### Immunofluorescence

To visualize the antigen uptake, 3 × 10^5^ JAWSII and RAW264.7 cells were seeded on a 14 mm glass coverslip, respectively. 5 μg OVA alone or with the same amount of Diprovocim was subjected to the cells and incubated for 24 h. PBS acted as the negative control. Cells were fixed with 4% PFA for 20 min at 4 °C and stained with DAPI (Sigma-Aldrich, D9542, 1:500). Coverslips were mounted with ProLong Gold Antifade Mountant (Thermo Fisher, P10144) and imaged using a confocal fluorescence microscope (Olympus, FV-3000).

### Histopathology

Lung tissues were dissected from 8 h, 1 day and 3 days after nasal injection and fixed with 4% PFA. Lung tissues were embedded in paraffin, sectioned into 4 µm-thick, and stained with hematoxylin and eosin. Histopathological analysis assessed eosinophil infiltrates, epithelium damage, and necrosis in each mouse, as previously described^87^. The number indicates the number of mice with (+) or without (−) eosinophil infiltrates, epithelium damage or necrosis.

### Tissue processing

C57BL/6 mice, 6–8 weeks old, were anesthetized by the inhalation anesthetic isoflurane (RWD, R510) at 5% for anesthetic induction and 2% for maintenance in oxygen using a precision vaporizer with induction chamber and waste gas scavenger. To analyze the immune responses in vivo, mice were immunized at two-week intervals. At the end point of experiments, mice were euthanized by inhalation isoflurane overdose (>5%) followed by cervical dislocation. Peripheral blood was collected 7 days after each immunization, with the exception of the first immunization, for which blood was collected 14 days following. The peripheral blood was lysed with ACK lysis buffer (Biosharp, BL503A) twice, and washed with PBS containing 1% FBS. For the dendritic cell subtype classification and activated evaluation, MLNs and lungs were collected 24 h after injection, and processed into single-cell suspensions for analysis by flow cytometry. Specifically, the lungs were minced into 1 mm^2^ pieces, digested with 1 ml of collagenase D (2 mg/ml)/DNase I (5 mg/ml), both from Roche, at 37 °C for 60 min, and then passed through 40 μm cell strainers^88^. Single-cell suspensions of lymph nodes were prepared by passing the tissues through 40 μm cell strainers directly. The remaining cells were washed with PBS + 1% FBS.

### Phagocytosis experiment in vivo

Previously anesthetized mice were intranasally immunized with 30 μg OVA-Cy5, with or without 30 μg Diprovocim or 10 μg Pam2CSK4. Mice were sacrificed 24 h later, and lung cells and MLNs were collected. Next, lung cells were incubated with FITC-anti-CD11c (1:100, BioLegend, 117305), PE-anti-CD11b (1:100, BioLegend, 101207), BV421-anti-CD24 (1:100, BioLegend, 101825). MLNs were incubated with FITC-anti-CD11c (1:100, BioLegend, 117305), PE-anti-CD11b (1:100, BioLegend, 101207). All stained cells were examined by flow cytometry and analyzed by FlowJo v10.8.

### Mice immunization and flow cytometry

Previously anesthetized mice were intranasally (i.n.) or intramuscularly (i.m.) immunized with 30 μg OVA or 30 μg RBD-Fc, with or without 30 μg Diprovocim or 10 μg Pam2CSK4. For protein vaccines, OVA was purchased from Sigma-Aldrich (A5503). pFUSE-RBD-hIgG1-Fc2 was kindly provided by Dr. Zezhong Liu from Fudan University. The plasmids were transfected into 293 F cells, leading to the expression of RBD-Fc. The protein was purified using Protein A + G Agarose (P2019, Beyotime). For the CD86 marker, lung cells were incubated with FITC-anti-CD11c (1:100, BioLegend, 117305), PE-anti-CD11b (1:100, BioLegend, 101207), APC-anti-CD24 (1:100, BioLegend, 138505), and BV421-anti-CD86 (1:100, BioLegend, 105032). For the CD40 marker, lung cells were incubated with FITC-anti-CD11c (1:100, BioLegend, 117305), PE-anti-CD11b (1:100, BioLegend, 101207), BV421-anti-CD24 (1:100, BioLegend, 101825), and APC-anti-CD40 (1:100, BioLegend, 124611). For MLNs, cells were incubated with FITC-anti-CD11c (1:100, BioLegend, 117305), PE-anti-CD11b (1:100, BioLegend, 101207), BV421-anti-CD86 (1:100, BioLegend, 105032) and APC-anti-CD40 (1:100, BioLegend, 124611). To evaluate intracellular cytokine staining, the PBMCs were stimulated with 8 μg/ml OVA_323-339_ (ISQAVHAAHAEINEAGR, Syneptide Co., Ltd) and OVA_257-264_ (SIINFEKL, Syneptide Co., Ltd), or 2 μg/ml SARS-CoV-2 spike peptide pool (Sinobiological, PP003) for 16 h in the presence of anti-CD28 antibody (BioLegend, 102102, 4 μg/ml). Brefeldin A (BioLegend, 420601) was added to the cell 4 h after cell culture. Cells treated with anti-CD28 and Brefeldin A without peptide stimulation served as a negative control. Cells treated with PMA (MultiSciences, 70-CD0001, 1:1000) plus Ionomycin (MultiSciences, 70-CD0002, 1:1000), anti-CD28 and Brefeldin A without peptide stimulation were served as a positive control. Cells were stained with Zombie Aqua™ Fixable Viability Kit (1:400, BioLegend, 423101) to label dead cells. Cells were blocked by 10 μg/ml anti-CD16/CD32 antibody (BioLegend, 101302) for 20 min, incubated with FITC-anti-CD3 (1:100, BioLegend, 100203), PE-anti-CD4 (1:100, BioLegend, 100407) and BV711-anti-CD8a (1:100, BioLegend, 100747) antibodies for 30 min on ice, fixed and permeabilized by Fix/Perm solution (BD, 554714) for 20 min on ice. Cells were washed and suspended in Perm/Wash buffer (BD, 554723) containing BV421-anti-IFN-γ (1:100, BioLegend, 505829), APC-anti-TNF-α (1:100, BioLegend, 506307) for 1 h on ice. For the evaluation of cells for flow cytometry, all the gating strategies are shown in Supplementary Fig. 2. All stained cells were examined by flow cytometry and analyzed by FlowJo v10.8.

### ELISA

Peripheral blood was collected from immunized mice at indicated time, kept at room temperature for 1 h, and centrifuged at 3000 × *g* for 20 min to collect serum. To collect BALF, mice were perfused thoroughly with ice-cold PBS followed by intratracheal lavage with 0.5% BSA in PBS 7 days after the last injection. An indirect ELISA was performed to determine antibody titers against OVA or WT RBD. A 96-well plate was coated with 1 μg of OVA or 0.1 μg RBD protein per well at 4 °C overnight. The plates were washed with PBST (0.05% Tween-20 in PBS) and incubated with 5% BSA in PBST for 1 h at room temperature. Mouse serum was initially diluted 1:50 in PBST and then serially diluted 5-fold. BALF was initially diluted 1:4 in PBST and then serially diluted 4-fold. Diluted serum and BALF samples were incubated with the coated plates for 1 h at room temperature. After washing, HRP-conjugated anti-mouse IgG (Cell signaling Technology, 7076, 1:3000), and HRP-anti-mouse IgA (SouthernBiotech, 1040-05, 1:4000) antibodies was respectively incubated for 1 h. After washing, the titers of specific antibody subtypes were quantified by using 3,3’,5,5’-tetramethylbenzidine (TMB, Beyotime, P0209) as the substrate and reading the reaction at OD450 by the Varioskan Lux Microplate Reader (Thermo Fisher). Endpoint titers were expressed as the highest reciprocal serum dilution exhibiting OD450 value that was 2.1-fold over the background.

### Pseudovirus neutralization assay

To generate pseudoviruses with S protein of WT, Delta, BA.1, or BA.5 strains, pcDNA3.1-spike (WT, Delta, BA.1 or BA.5), psPAX2 and pLenti-CMV-Puro-Luc (168w-1) plasmids were co-transfected to HEK293T using Lipo293 according to the manufacturer’s instruction. The culture supernatants containing Luc-expressing pseudoviruses were harvested after 72 h and stored at −80 °C until use. The hACE2-293T cells (2 × 10^4^ cells/well) were seeded in the black flat-bottom 96-well plates. Inactivated sera were first diluted 25-fold then 4-fold serially diluted in DMEM, and subsequently co-incubated with the same volume of pseudovirus for 1 h at 37 °C. The mixtures were subjected with 10 μg/mL polybrene (Beyotime, C0351) to hACE2-293T cells for 6-h absorption. The mixtures were discarded, and fresh culture medium was added and incubated for an additional 42 h at the cell incubator. After incubation, the infected cells were lysed using firefly luciferase lysis buffer (Beyotime, RG126M), and the luciferase substrate (Beyotime, RG058M) was applied for the luciferase assay. The relative light unit (RLU) was measured using the Varioskan Lux Microplate Reader. The half pseudovirus neutralization titers (PVNT_50_) were determined with a four-parameter nonlinear regression curve by GraphPad Prism 10.3.1.

### Biolayer Interferometry

The monoclonal antibodies were kindly provided by Dr. Teng Zuo at Hefei Institutes of Physical Science and were labeled with biotin (Elabscience, E-LK-B002). Affinity assays were performed and analyzed using BLI on an Octet® R8 (Sartorius, German) at ambient temperature with shaking at 1000 rpm. Monoclonal antibodies of P2C-1F11 and COV2-2196 were diluted to 3 μg/ml in buffer (0.02% PBST). RBD-Fc or Diprovocim+RBD-Fc were diluted to 66.7 nM in 0.02% PBST in PBST and then serially diluted 3-fold for a final concentration of 0.274 nM. Reagents were applied to a black 96-well Greiner Bio-one microplate at 200 μl per well as described below. Antibodies were immobilized onto Octet® SA biosensors (Sartorius, German) using the following sensor incubation times. Octet® SA biosensors were hydrated in water for 10 min, and were then equilibrated in buffer for 60 s. The tips were loaded with antibodies for 120 s and washed with buffer for 120 s. The association step was performed by dipping the SA biosensors into diluted antigen for 180 s, then dissociation was measured by inserting the biosensors back into buffer for 300 s. The data were baseline subtracted and the plots fitted using the Octet BLI Analysis 12.

### Statistical analysis

All statistical analyses were performed using GraphPad Prism 10.3.1. Data were shown as mean ± SEM as indicated in the experiments, except for antibody titers, which are presented as geometric mean ± 95% CI. Comparisons of two groups were assessed using two-tailed Student’s *t* test. Comparisons of multiple groups were assessed using One-way ANOVA with Tukey’s multiple comparisons test, Kruskal–Wallis test, two-way ANOVA with Tukey’s multiple comparisons test, or two-way ANOVA with Dunnett’s multiple comparisons test.

## Supplementary information

Supplementary information

## Data Availability

All data supporting the findings of this study are available within the paper and supplementary information.
