# Supplementary material for: Self-assembling TLR2 agonists promote mucosal immune responses without pulmonary immunopathologic injuries in mice
Source: NPJ Vaccines. 2025 Jun 18;10:127. doi: 10.1038/s41541-025-01185-y (PMC12177044; doi:10.1038/s41541-025-01185-y)
Supplement: Supplementary file 1 — Supplementary information [file 41541_2025_1185_MOESM1_ESM.pdf]

Supplementary material

Self-assembling TLR2 agonists promote mucosal immune responses without pulmonary immunopathologic injuries in mice

Zhangping Huang[1,2], Caiguanxi Deng[2], Lin Peng[2], Liru Shang[2], Juan Jiang[2], Wei Yu[2], Hao Yang[2], Jing Liu[2], Liwei Jiang[3], Teng Zuo[3], Ji Wang[2*], Xiafeng Wang[2*]

Supplementary Figure 1:

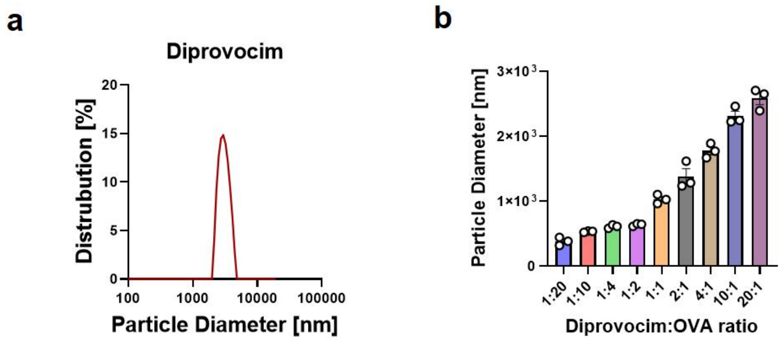

Supplementary Figure 1. Particle sizes of Diprovocim self-assembling particles.

a Particle diameters of Diprovocim self-assembling particles. **b** Particle diameters of self-assembling particles formed by mixing Diprovocim and OVA at different Ratios (Diprovocim:OVA). n=3.

Supplementary Figure 2:

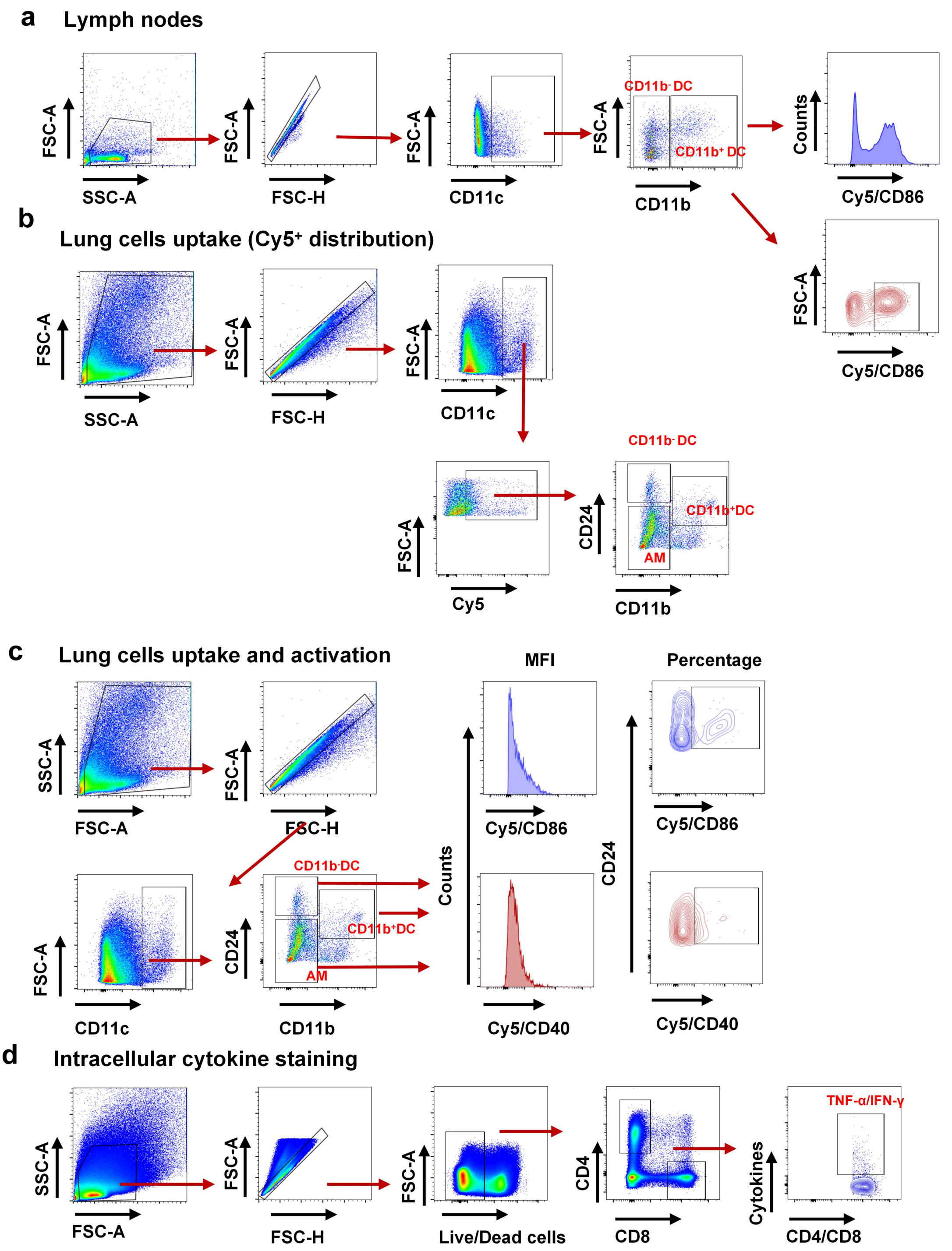

Supplementary Figure 2. Gating strategies of experiments in mice.

a Gating strategies of cellular uptake and activation in MLNs. **b** Distribution of $Cy5^+$ $CD11c^+$ cells in lungs. **c** Gating strategies of cellular uptake and activation in lungs. **d** Gating strategies of intracellular cytokine staining of IFN-γ and TNF-α.

Supplementary Figure 3:

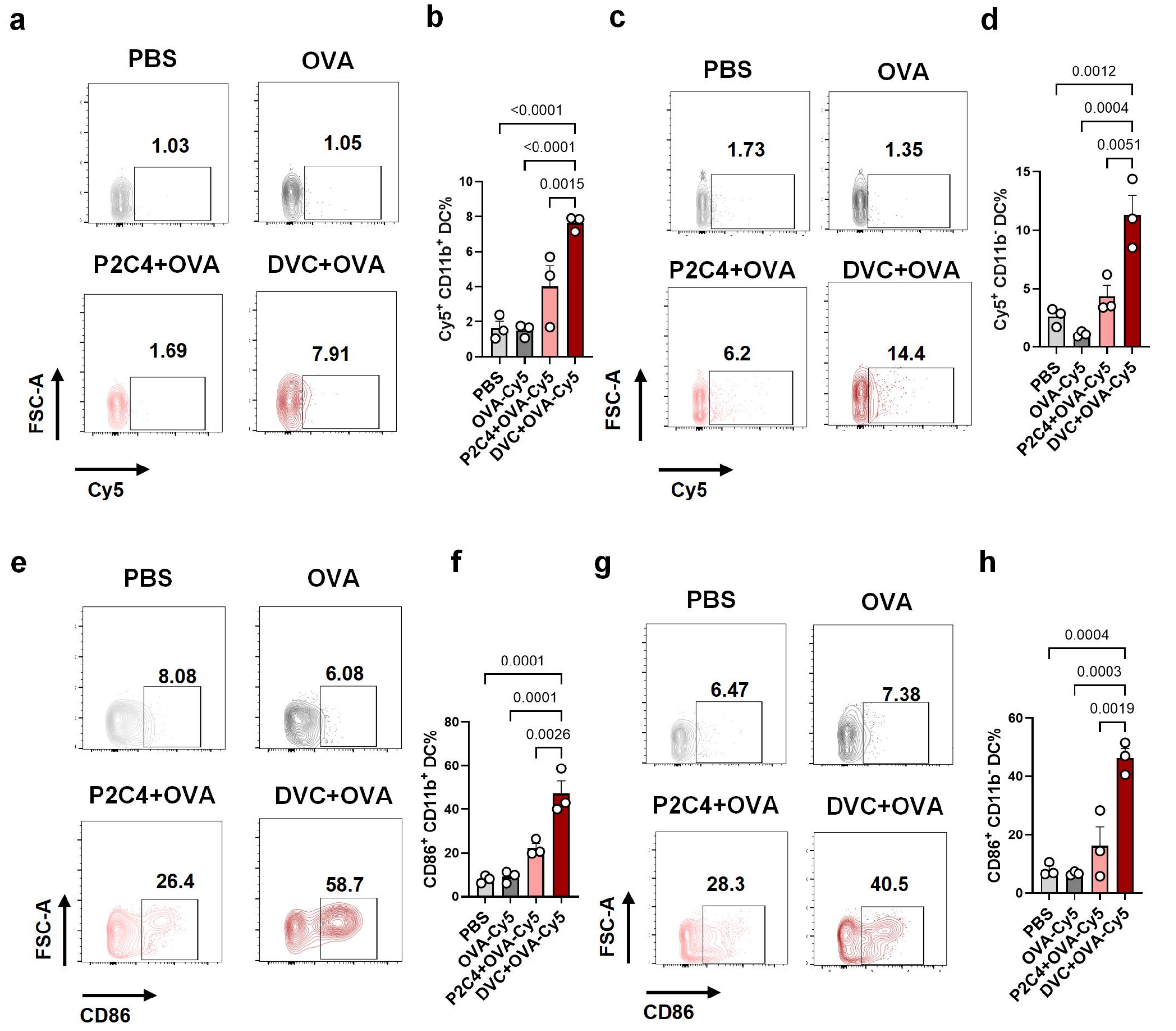

Supplementary Figure 3. Cellular uptake and activation of cells in MLNs 24 h post nasal immunization.

a-d The percentage of Cy5$^+$ CD11b$^+$ and Cy5$^+$ CD11b$^-$ DC in MLNs.n=3. **e-h** The percentage of CD86$^+$ CD11b$^+$ and CD86$^+$ CD11b$^-$ DC in MLNs. n=3. Data are shown as mean ± SEM. Statistical significance was determined using one-way ANOVA with Tukey's multiple comparisons test.

Supplementary Figure 4:

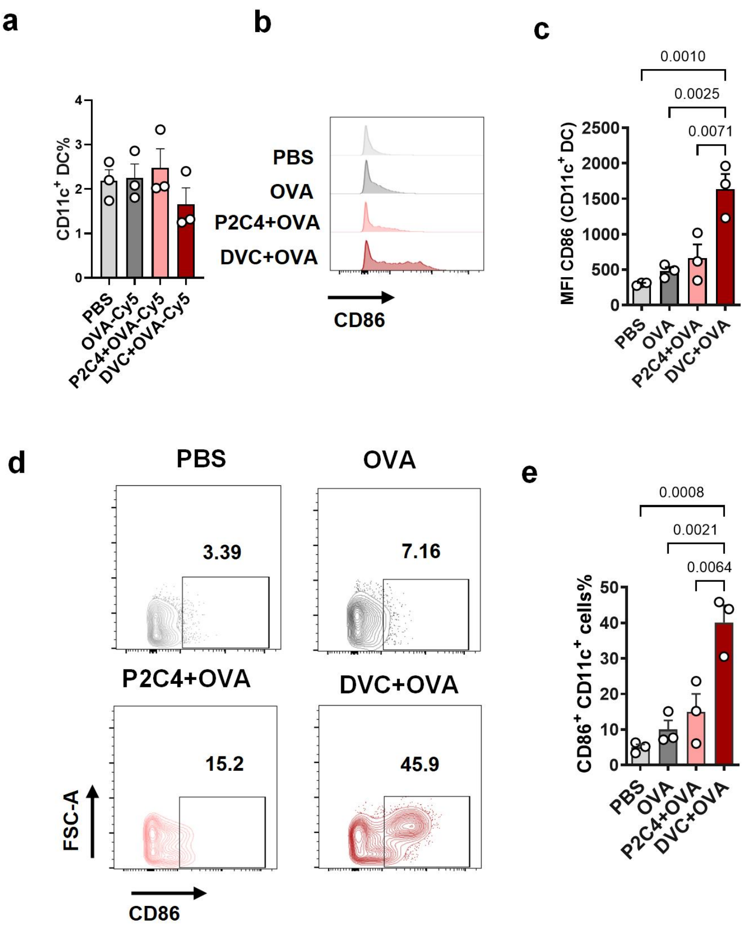

Supplementary Figure 4. Activation of CD11c$^+$ cells in MLNs 24 h post nasal immunization.
a The percentage of CD11c$^+$ cells in MLNs. n=3. **b-e** The MFI of CD86 in CD11c$^+$ cell and the percentage of CD86$^+$ CD11c$^+$ in MLNs. n=3. Data are shown as mean ± SEM. Statistical significance was determined using one-way ANOVA with Tukey's multiple comparisons test.

Supplementary Figure 5:

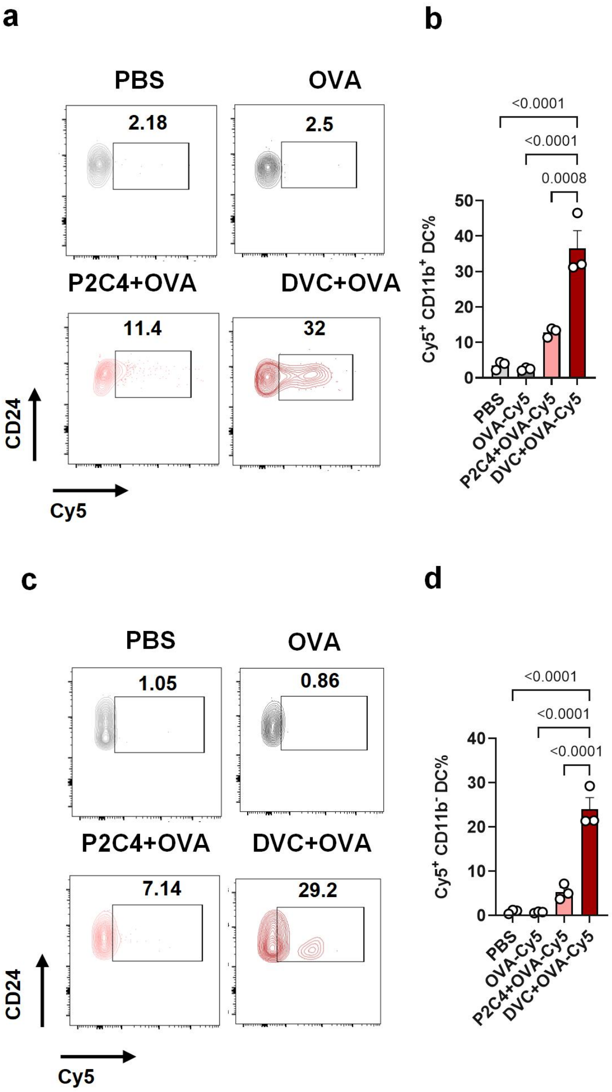

Supplementary Figure 5. Cellular uptake in lungs 24 h post nasal immunization.

a-d The percentage of $Cy5^{+}$ $CD11b^{+}$ and $Cy5^{+}$ $CD11b^{-}$ DC in lungs. n=3. Data are shown as mean ± SEM. Statistical significance was determined using one-way ANOVA with Tukey's multiple comparisons test.

Supplementary Figure 6:

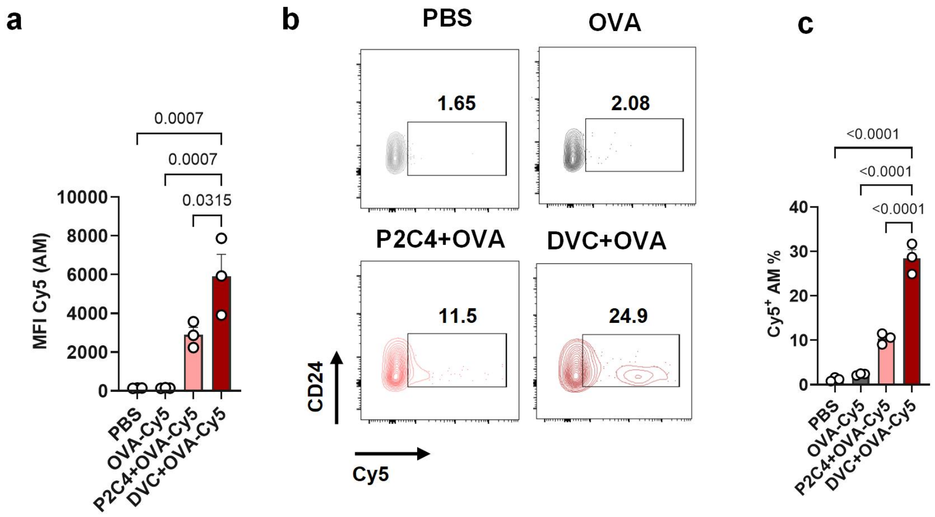

Supplementary Figure 6. Cellular uptake and activation of alveolar macrophage (AM) in lungs 24 h post nasal immunization.

a-c The MFI of Cy5 in AM (**a**) and the percentage of $Cy5^+$ AM (**b, c**) in lungs. n=3. Data are shown as mean ± SEM. Statistical significance was determined using one-way ANOVA with Tukey's multiple comparisons test.

Supplementary Figure 7:

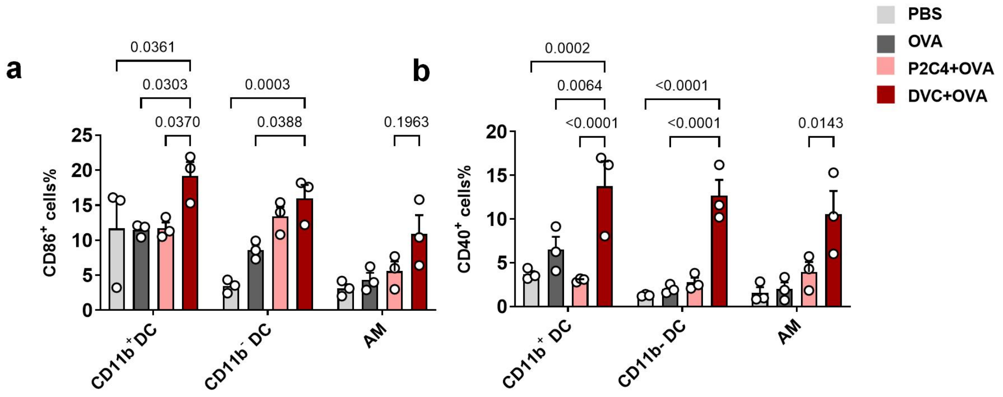

Supplementary Figure 7. Activation of cells in lungs 24 h post nasal immunization.

a, b Percentages of CD86 (**a**) and CD40 (**b**) in different cell types of lungs were monitored. n=3. Data are shown as mean ± SEM. Statistical significance was determined using two-way ANOVA with Tukey's multiple comparisons test.

Supplementary Figure 8:

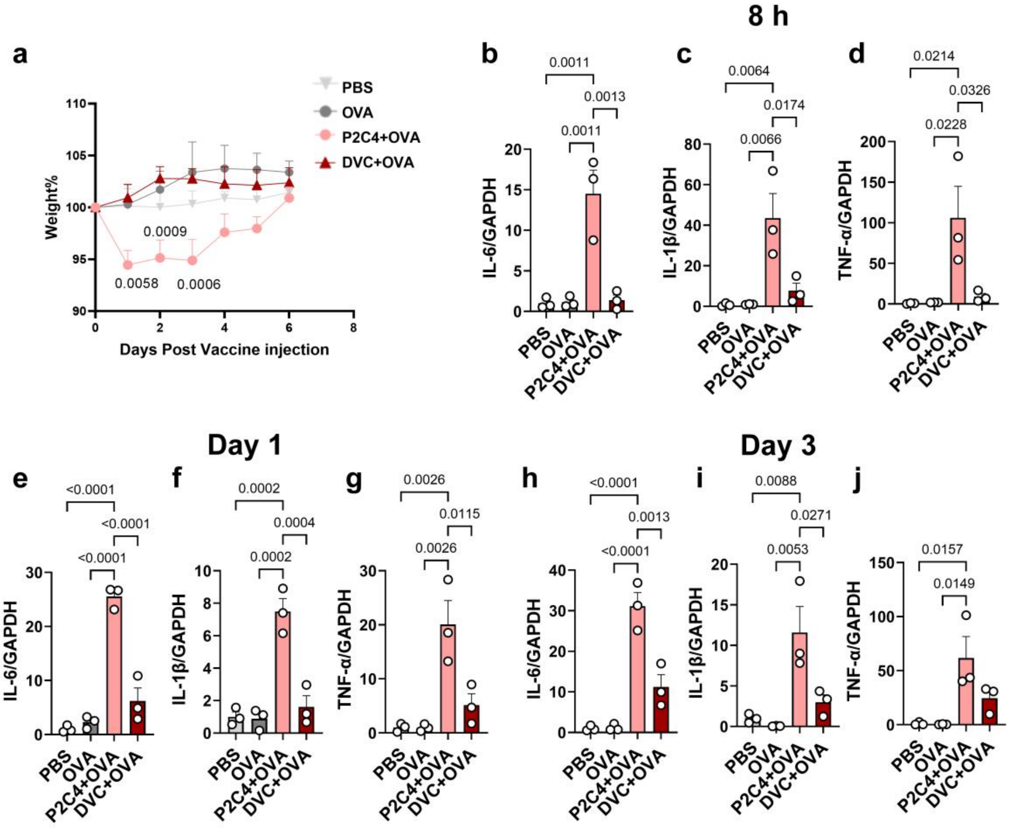

Supplementary Figure 8. The repeat experiments of Diprovocim eliciting a reduced inflammatory profile.

a Body weight changes were monitored for 6 days post-immunization. n=5. **b-j** Relative mRNA expression levels of IL-6, IL-1β, and TNF-α in lung tissue were assessed at various time points post-nasal vaccination by RT-qPCR. n=3. Data are shown as mean ± SEM. Statistical significance was determined using two-way ANOVA with Dunnett's multiple comparisons test **(a)** or one-way ANOVA with Tukey's multiple comparisons test **(b-j)**.

Supplementary Figure 9:

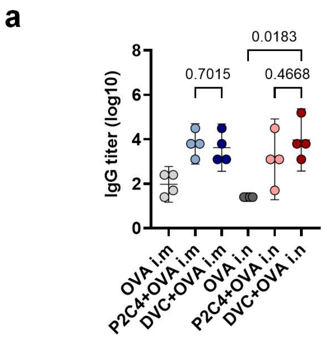

Supplementary Figure 9. Self-assembling particles vaccination elicited long-term antibody responses.

a OVA-specific IgG antibody titer in serum on day 60 after the second dose. n=4. Data are shown as geometric mean ± 95% CI. Statistical significance was determined using Kruskal-Wallis test.

Supplementary Figure 10:

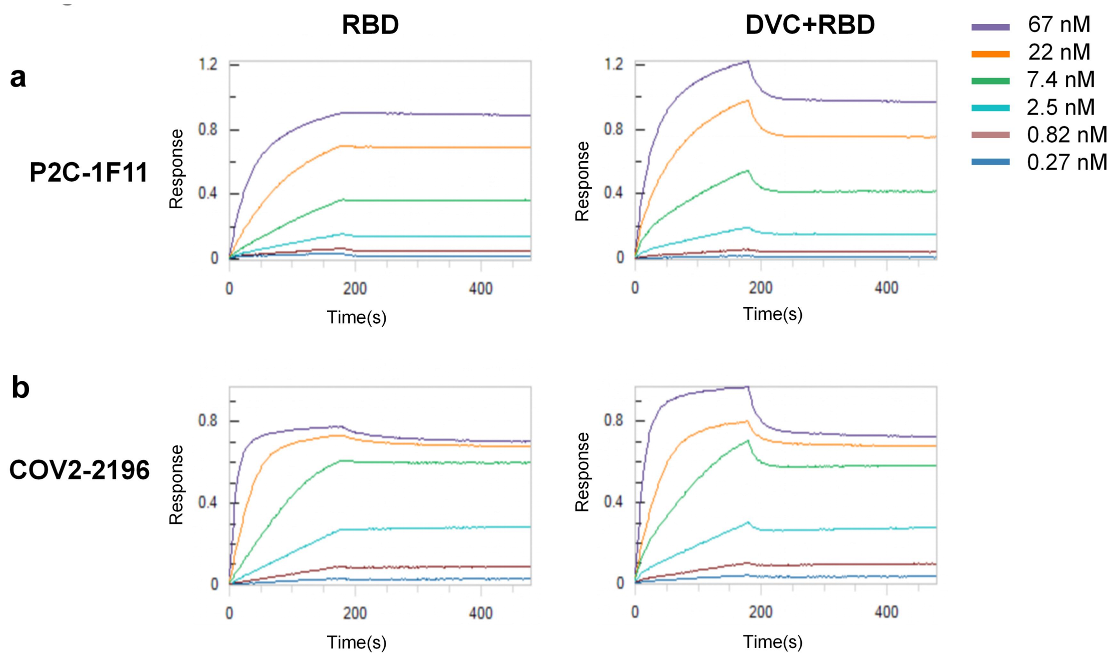

c

Monoclonal Antibodies	Dissociation constant (KD, Kon/Koff, nM)	Association rate constant (Kon, $M^{-1} \cdot s^{-1}$)	Dissociation rate constant (Koff, s^{-1})
P2C-1F11 (RBD)	0.27	9.75×10^{5}	2.61×10^{-4}
P2C-1F11 (DVC+RBD)	0.82	1.69×10^{6}	1.38×10^{-3}
COV2-2196 (RBD)	0.17	1.21×10^{6}	2.07×10^{-4}
COV2-2196 (DVC+RBD)	0.59	1.25×10^{6}	7.43×10^{-4}

Supplementary Figure 10. The binding affinity of monoclonal antibodies with RBD-Fc or Diprovocim+RBD-Fc using Biolayer Interferometry (BLI).

a, b Binding curves of monoclonal antibodies (P2C-1F11, COV2-2196) to RBD or DVC+RBD. **c** Binding affinities between RBD and antibodies were summerized in **(c).**

Supplementary Table 1

Treament	Eosinophil infiltrates		Alveolar damage		Necrosis	
	+	−	+	−	+	−
PBS		4		4		4
OVA (1)		4		4		4
OVA (3)		4		4		4
DVC+OVA (1)		4		4		4
DVC+OVA (3)		4		4		4
P2C4+OVA (1)	4		4		4	
P2C4+OVA (3)	4		4		4	

Histopathological analysis of pulmonary immunization

Eosinophil infiltrates, epithelium damage, and necrosis of each mouse in different groups were analyzed.. The number indicates the number of mice with (+) or without (−) eosinophil infiltrates, epithelium damage or necrosis. n=4.
